# Letter to the Editor: Long‐Term Effectiveness of a Stent‐Less Strategy With Drug‐Coated Balloon in Coronary Artery Disease: 3‐Year Follow‐Up

**DOI:** 10.1002/clc.70236

**Published:** 2025-12-19

**Authors:** Hamza Rashid

**Affiliations:** ^1^ Nowshera Medical College Nowshera Pakistan

To the Editor,

I read with great interest the article by Meunier et al. reporting the 3‐year outcomes of a stent‐less strategy (SLS) that used scoring balloon lesion preparation followed by paclitaxel‐coated balloon angioplasty (DCB), with a bailout drug‐eluting stent (DES) only when required [1]. The prospective, all‐comers design and the long follow‐up provide valuable real‐world data on this evolving “leave‐nothing‐behind” approach, extending earlier 1‐year evidence from the same group [2].

The authors are to be commended for proposing a practical algorithm in routine care and for introducing the “metal index” as a simple way to capture stent burden. The high proportion of patients eligible for SLS (≈85%) and the very low 3‐year target lesion revascularization (TLR) rate in the DCB‐only group (2.6%) are encouraging findings.

Some points, however, deserve clarification. First, patients in the bailout‐DES group had longer lesions and more complex anatomy (multivessel disease, chronic total occlusion, bifurcation lesions). These are well‐known predictors of adverse outcomes, and residual confounding may explain their higher TLR rates. Second, the main analysis relied on unadjusted Kaplan–Meier curves; a multivariable Cox model adjusting for lesion length, clinical presentation, left ventricular function, and lesion type would help clarify whether the metal index or SLS independently predicts TLR. Third, antiplatelet therapy was heterogeneous and incompletely described; given that prolonged dual therapy was common even in DCB patients, reporting both ischemic and bleeding outcomes is essential. Finally, ~8% of patients were lost to follow‐up; sensitivity analyses would increase confidence in the results.

Comparable long‐term results with “less DES” strategies have been described in chronic total occlusion cohorts, supporting the concept of minimizing stent burden when feasible [3].

In summary, the SLS algorithm is promising, safe, and associated with low 3‐year TLR rates. Larger multicenter randomized trials with standardized lesion preparation and prespecified bleeding endpoints are needed to confirm these findings.

Sincerely,

## Data Availability

The author has nothing to report.

## References

[1] L. Meunier , S. Eccleshall , R. Bakdi , et al., “Long‐Term Effectiveness of a Stent‐Less Strategy With Drug Coated Balloon in Coronary Artery Disease: 3‐Year Follow‐up of a Prospective All‐Comers Observational Study,” Clinical Cardiology 48, no. 8 (2025): e70189, 10.1002/clc.70189. 40745698 PMC12313834

[2] L. Meunier , M. Godin , G. Souteyrand , et al., “Prospective Single‐Centre Evaluation of the Safety and Efficacy of Percutaneous Coronary Interventions Following a Decision Tree Proposing a No‐Stent Strategy in Stable Patients With Coronary Artery Disease (SCRAP Study): 1‐Year Outcomes,” EuroIntervention 18 (2022): 2054–2064, 10.4244/EIJ-D-22-00214.

[3] X. Wang , X. Yang , W. Lu , et al., “Long‐Term Outcomes of Less Drug‐Eluting Stents by the use of Drug‐Coated Balloons in De Novo Coronary Chronic Total Occlusion Intervention: A Multicenter Observational Study,” Frontiers in Cardiovascular Medicine 10 (2023): 1045859, 10.3389/fcvm.2023.1045859. 36937919 PMC10022494

